# Supplementary material for: Chemokine Receptor Expression on T Cells Is Modulated by CAFs and Chemokines Affect the Spatial Distribution of T Cells in Pancreatic Tumors
Source: Cancers (Basel). 2022 Aug 6;14(15):3826. doi: 10.3390/cancers14153826 (PMC9367555; doi:10.3390/cancers14153826)
Supplement: Supplementary file 1 [file cancers-14-03826-s001.zip › cancers-1713334-supplementary.pdf]

Supplementary Table S1

Markers	Fluorochrome	Clone	Company	Catalogue #	Staining
**Lymphocyte's markers**					
CD3	BV510	UCHT1	BD	563109	EC
CD3	BV785	UCHT1	Biolegend	300472	EC
CD4	A700	RPA-T4	BD	557922	EC
CD8	APC-Cy7	SK1	BD	557834	EC
CXCR4	BV421	12G5	BD	562448	EC
CXCR3	APC	1C6	BD	550967	EC
CCR5	PECF594	2D7	BD	562456	EC
7AAD	7AAD	-	BD	559928	

Antibodies used to characterize lymphocytes in flow cytometer. Abbreviations; **CD**, Cluster of differentiation, **CXCR,** Chemokine (C-X-C) motif receptor, **CCR**, Chemokine (C-C) motif receptor. Companies; **BD**, BD Biosciences (Franklin Lakes, NJ, USA), **BioLegend**, Biolegend (San Diego, CA, USA). Stainings; **EC**, Extracellular.

Supplementary Figure S1

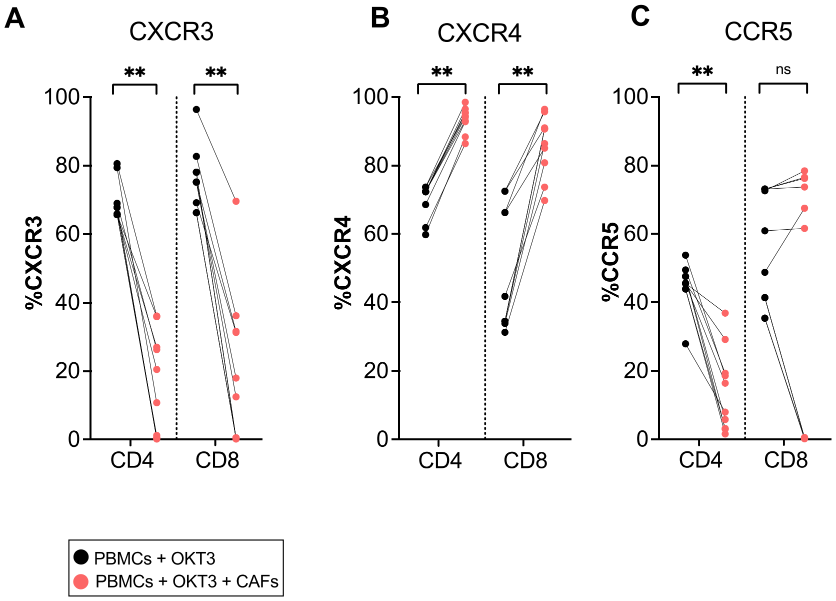

Supplementary Figure S1. CAFs modulate chemokine receptor expression on activated T cells. PBMCs were co-cultured with the absence (black dots) or presence (light red dots) of CAFs and activated with OKT3 (25ng/ml) for 5 days. Expression of **(A)** CXCR3, **(B)** CXCR4, and **(C)** CCR5 in CD4$^+$ and CD8$^+$ T cells. Dots and lines show paired samples. Wilcoxon signed-rank test was used to detect statistically significant differences between paired samples. ns, not significant, $**p < 0.01$.

Supplementary Figure S2

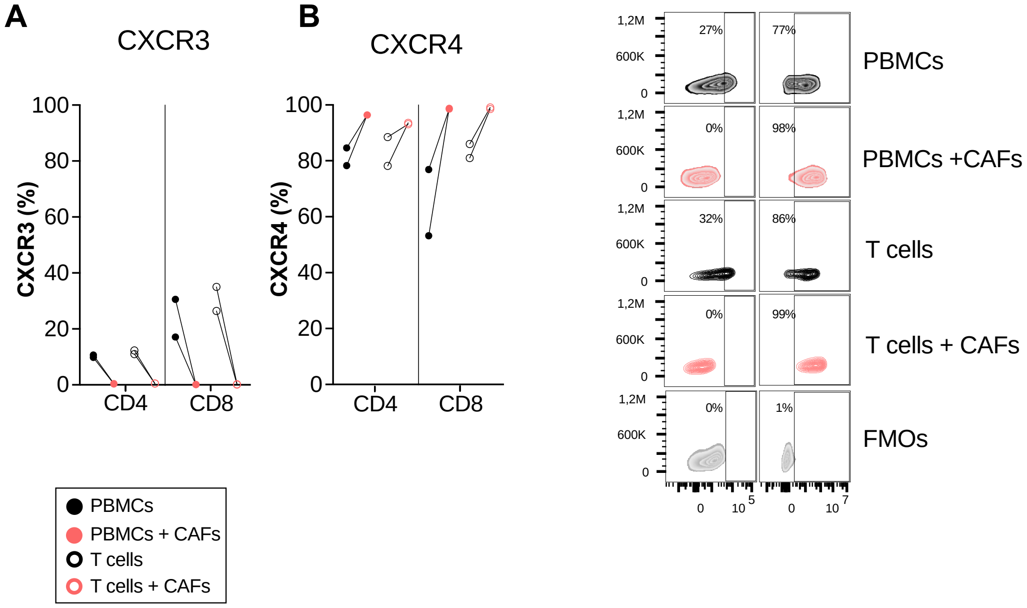

Supplementary Figure S2. CAFs modulate chemokine receptor expression on isolated T cells. PBMCs (filled circles) or isolated T cells ($CD3^+$) (open circles) were co-cultured in the absence (black) or presence (light red) of CAFs. Expression of **(A)** CXCR3 and **(B)** CXCR4. (right) Representative zebra plots showing the gating strategy in **(A-B)**.

Supplementary Figure S3

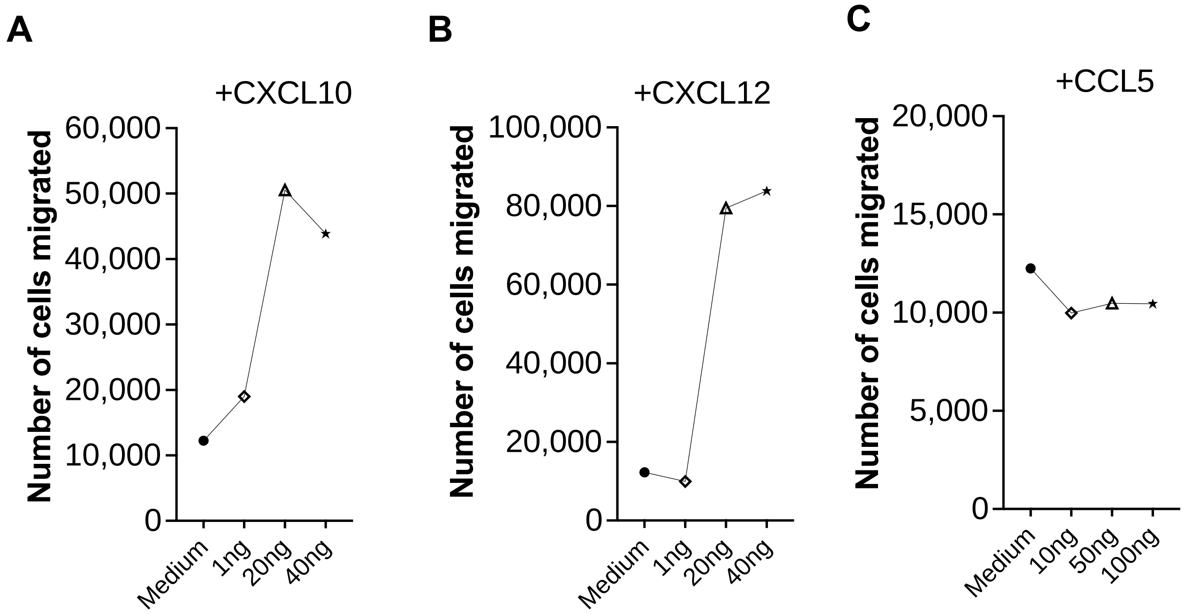

Supplementary Figure S3. T cells migrate towards CXCL10, CXCL12 but not CCL5. Number of migrated $CD3^+$ T cells towards **(A)** CCL5 (10ng, 50ng, 100ng) **(B)** CXCL10 (1ng, 20ng, 40ng) and **(C)** CXCL12 (1ng, 20ng, 40ng).

Supplementary Figure S4

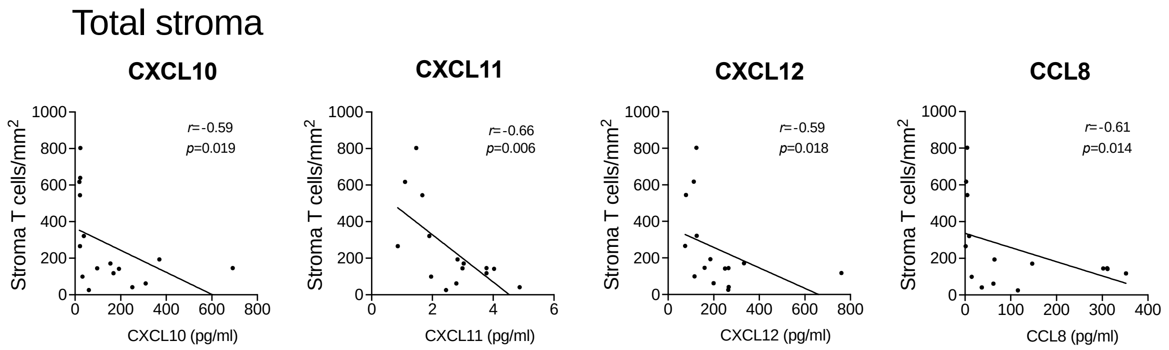

Supplementary Figure S4. Correlations between T cell number and chemokines. Correlations between the numbers of CD8^{+} T cells in tumor/stroma areas and chemokine secretion. Spearman *r* and *p*-values are presented. ns, not significant. $^{*}p < 0.05$, $^{**}p < 0.01$, $^{***}p < 0.001$.

Supplementary Figure S5

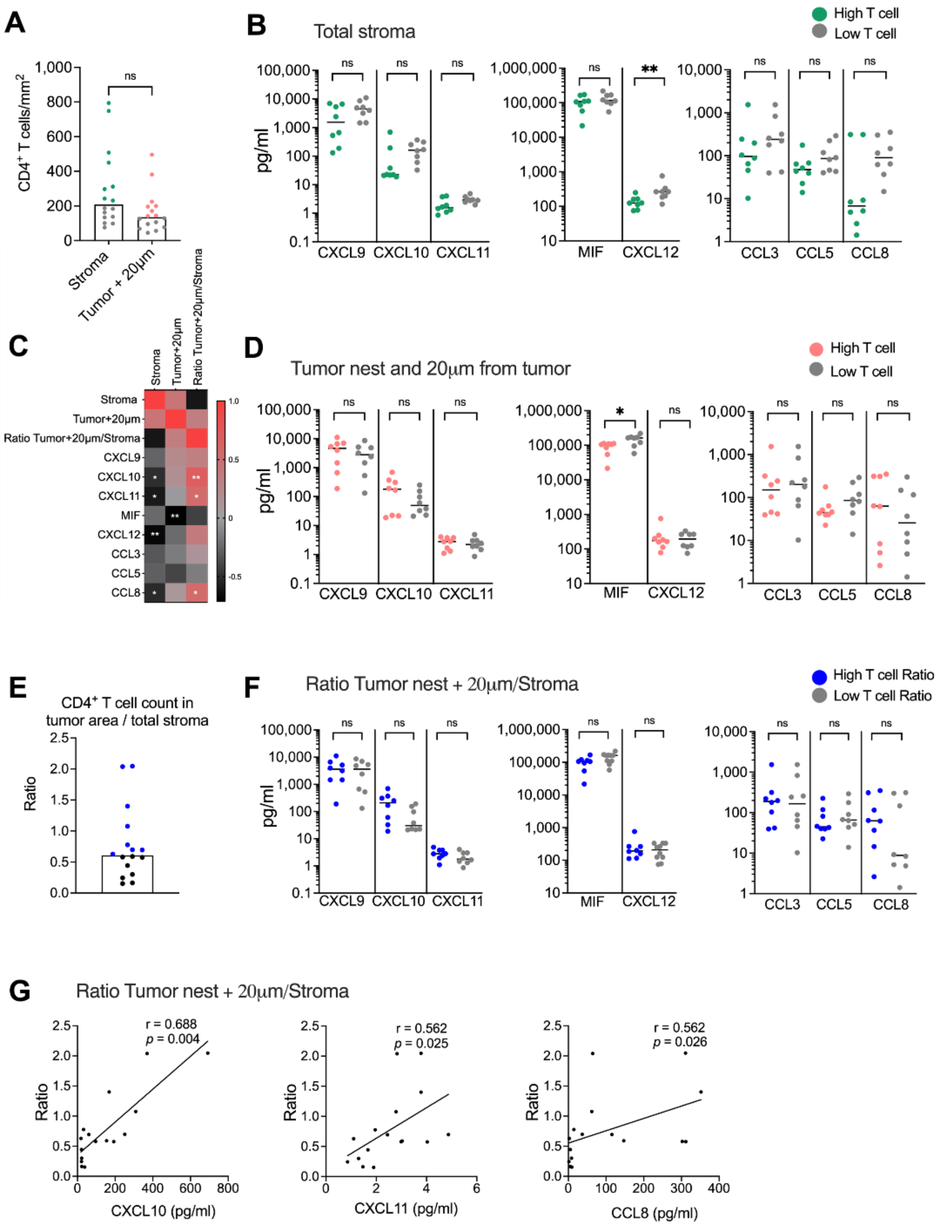

Supplementary Figure S5. Spatial localization of CD4⁺ T cells. (A) Number of CD4⁺ T cells in stroma and in the tumor nest + 20 µm from the tumor nests. Green and red dots show the donors with CD4⁺ T cells numbers above the median and grey dots show the donors with CD4⁺ T cells numbers below the median. **(B)** Secretion of chemokines in tissues with high CD4⁺ T cell numbers (green) and low CD4⁺ T cell numbers (grey). **(C)** Heat map correlation matrix showing positive (red) and negative (black) correlations between number of CD4⁺ T cells and chemokine secretion. **(D)** Secretion of chemokines in tissues with high CD4⁺ T cells numbers (red) and low CD4⁺ T cells numbers (grey) in the tumor nests + 20 µm from the tumor nests. **(E)** Ratio between CD4⁺ T cells in tumor nest + 20 µm from them. **(F)** Secretion of chemokines in tissues with high CD4⁺ T cell ratio (blue) and low CD4⁺ T cells ratio (grey). **(G)** Correlations between ratio of CD4⁺ T cells in tumoral areas (tumor nest + 20µm from tumor/total stroma) and CXCL10, CXCL11 and CCL8. Man-Whitney test was used to detect statistically significant differences between unpaired samples. Correlations were evaluated by using Spearman's correlation test. Spearman *r* and *p*-values are presented. ns, not significant. *$p < 0.05$, **$p < 0.01$, ***$p < 0.001$.
